# Glucagon-like Peptide-1 Receptor Agonists for the Treatment of Type 2 Diabetes in Youth

**DOI:** 10.17925/EE.2023.19.1.38

**Published:** 2023-05-23

**Authors:** Casey Berman, Alaina P Vidmar, Lily C Chao

**Affiliations:** 1. Division of Endocrinology, Department of Pediatrics, Children’s Hospital Los Angeles, Los Angeles, CA, USA; 2. Keck School of Medicine of University of Southern California, Los Angeles, CA, USA

**Keywords:** Diabetic nephropathy, glucagon-like peptide-1, glucagon-like peptide-1 receptor agonist, hyperglycaemia, obesity, paediatrics, type 2 diabetes

## Abstract

Glucagon-like peptide-1 receptor agonists (GLP-1RAs) have gained traction for the management of type 2 diabetes and obesity. Unlike several classes of antidiabetic medications that contribute to weight gain, GLP-1RAs not only reduce haemoglobin A1c, but also promote weight loss. While there is a large body of evidence supporting its safety and efficacy in adults, paediatric clinical trial data have only emerged in recent years. This review will discuss the limited treatment options for paediatric type 2 diabetes and the mechanism of action of GLP-1RAs as it pertains to physiological pathways relevant for type 2 diabetes, obesity and their related comorbidities. The outcomes of paediatric trials evaluating liraglutide, exenatide, semaglutide and dulaglutide in paediatric type 2 diabetes and obesity will be closely examined, including differences compared with adult studies. Finally, potential barriers and strategies to expanding GLP-1RA access in adolescents will be discussed. Future studies are needed to determine if the cardio-and renal-protective benefits of GLP-1RAs apply to youth-onset type 2 diabetes.

The incidence of youth-onset type 2 diabetes (T2D) is increasing.^1,2^ Growing evidence has demonstrated that youth-onset T2D is rapidly progressive, with earlier onset of life-l imiting complications compared with adult-onset T2D.^3,4^ Initiation of effective treatment that can restore beta cell function is critical. Until 2019, metformin and insulin were the only medications approved by the United States Food and Drug Administration (FDA) for the treatment of youth with T2D. In 2019, liraglutide became the first glucagon-like peptide-1 receptor agonist (GLP-1RA) approved for youth with T2D, followed by exenatide in 2021 and dulaglutide in 2022.^5–7^ According to the American Diabetes Association and the International Society for Pediatric and Adolescent Diabetes’ current clinical practice guidelines for the management of youth-onset T2D, metformin monotherapy is the standard initial treatment for youth with T2D, once metabolic control is restored with insulin in those who present with ketosis and/or marked hyperglycaemia.^8^ However, data collected from the Treatment Options for Type 2 Diabetes in Adolescents and Youth (TODAY) study demonstrated early glycaemic failure on metformin monotherapy, with a median treatment failure time of 11.5 months.^9^ Escalation of treatment, including the initiation of insulin, is recommended.^10^

The recommendation to start insulin therapy following metformin failure in youth contrasts with the guidelines for adults with T2D.^11^ In the management guidelines for adults with T2D, insulin or sulphonylureas are only added to the regimen if other medications fail to achieve the goal haemoglobin A1c (HbA1c) to avoid additional weight gain, which could worsen insulin resistance and overall glycaemic control and cardiometabolic health.^11,12^ Despite multiple new pharmacotherapies to treat adult-onset T2D, as of 2023 there remains only five medications that are approved by the FDA for the treatment of youth-onset T2D. Accordingly, medications approved for adult T2D have been used off-l abel in this cohort to improve weight and glycaemic outcomes.^13,14^

Across the lifespan, there is growing interest in utilizing GLP-1RAs in the treatment of T2D, given the potential to control hyperglycaemia and promote weight reduction, thus addressing the underlying pathophysiology of T2D.^15–17^ GLP-1RAs stimulate postprandial insulin secretion, reduce glucagon secretion, delay gastric emptying and decrease appetite, leading to improvements in glycaemic control and weight reduction.^17^ Given the rapid progression of beta cell failure and development of complications, there is considerable interest in treating youth with new oral and injectable agents that have been approved for use in adults with T2D.^18,19^ The objectives of this review are to: (1) review the mechanism of action of GLP-1RAs in T2D; (2) summarize the use of GLP-1RAs in youth-onset T2D, highlighting recent data on the use of dulaglutide in this age group; (3) discuss accessibility of GLP-1RAs in the paediatric population; and (4) conclude by reviewing the challenges of conducting phase III randomized clinical trials of these medications in the paediatric population, and discussing a potential pathway to facilitate approval of these drugs for adolescents with T2D.^20^

**Figure 1:**
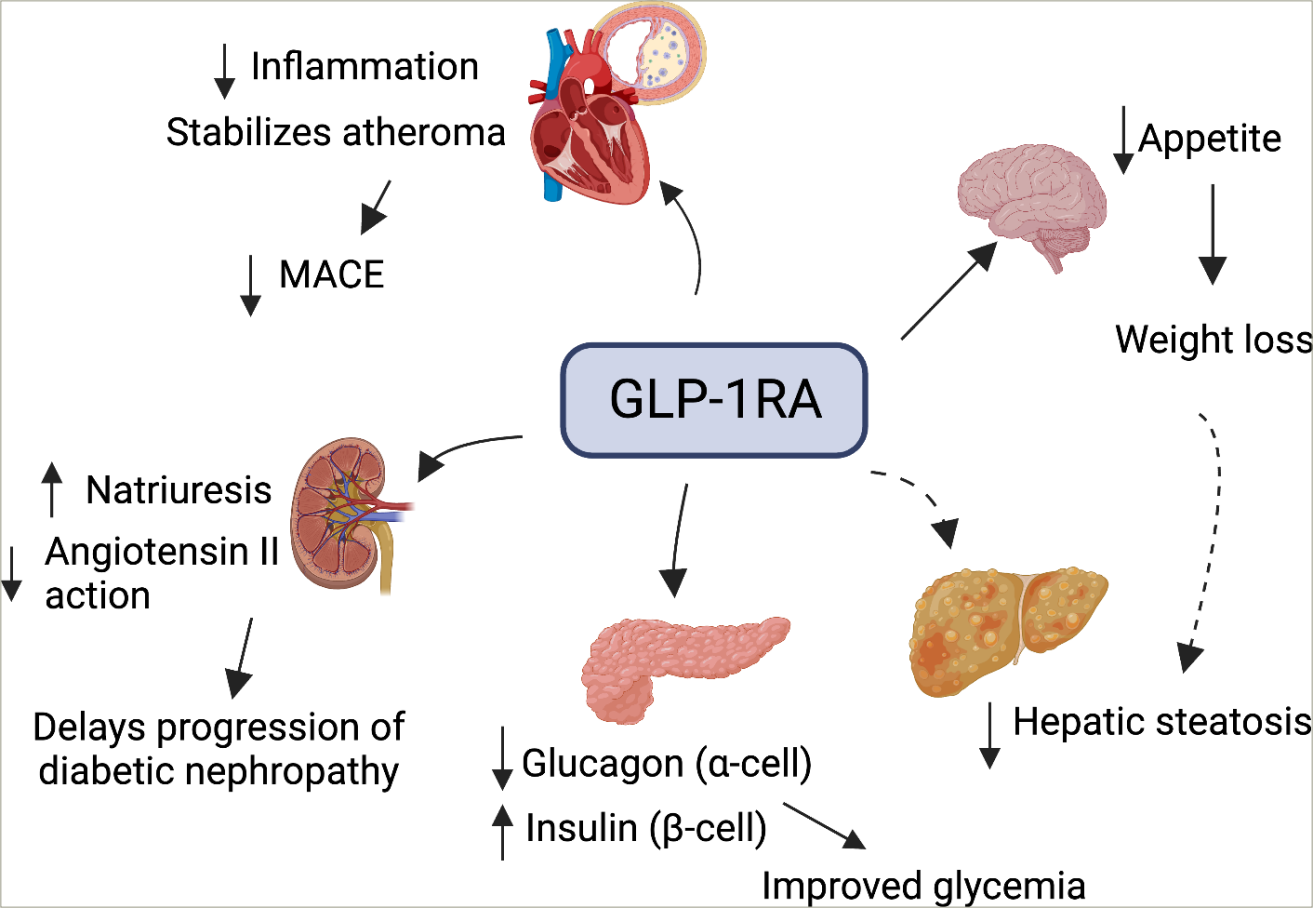
Therapeutic benefits of glucagon-like peptide-1 receptor agonists in obesity, diabetes and diabetes-related complications

*GLP-1RA = glucagon-like peptide-1 receptor agonist; MACE = major adverse cardiac events. Dashed arrow indicates indirect effect. Created with BioRender.com.*

## Glucagon-like peptide-1 mechanism of action

### Expression and regulation

Glucagon-like peptide-1 (GLP-1), which is encoded by the preproglucagon gene (*Gcg*), is a pleotropic hormone secreted by the intestinal L cells in response to nutrients.^21^ Proconvertase (PC) cleavage of the proglucagon molecule releases multiple peptides, including glucagon (aa 33–61), GLP-1 (aa 72–107/108) and GLP-2 (aa 126–158).^16^ This process occurs in a tissue-specific fashion to coordinate the activity of these hormones, which may have opposing functions. The expression of PC1 in the enteroendocrine L cells and the nucleus tractus solitarii (NTS) directs the cleavage of *Gcg* into GLP-1 (along with other peptides), whereas PC2 expression in the alpha cells results in the generation of glucagon.

GLP-1 is secreted by the distal ileum and colon and a population of neurones in the NTS, in response to nutrient and neural inputs.^22^ GLP-1 level is lowest during fasting and rapidly increases after feeding.^23^ Luminal exposure of the L cells to nutrients (carbohydrates, amino acids and lipids) induces GLP-1 secretion into the intestinal microvasculature to exert its humoral effects and stimulate enteric neurones. GLP-1 secretion is also stimulated by neurotransmitters from vagal and enteric neurones.^24^ In the brain, the NTS neurones are the primary source of endogenous GLP-1, whose secretion is stimulated by leptin and gastric distension. The downstream signalling through the GLP-1 receptor (GLP-1R) mediates the biological functions of GLP-1. The pleiotropic functions of GLP-1RA are described below (*Figure 1*).

### Rationale for use in type 2 diabetes

GLP-1RAs have become an attractive treatment option in T2D partly due to their glucose-dependent incretin effect. Binding of GLP-1 to the GLP-1R on the beta cell membrane activates cyclic adenosine monophosphate-mediated protein kinase A (PKA) signalling, resulting in exocytosis of the insulin granules.^22^ This coordinated process is highly dependent on the ambient glucose concentration, which minimizes the risk of hypoglycaemia in patients receiving GLP-1RA treatment.^22^ Conditional mouse models that alter tissue-specific expression of GLP-1Rs have shown that beta cell GLP-1R expression is required for hyperglycaemia-and GLP-1RA-i nduced insulin secretion.^25,26^ The importance of GLP-1R signalling in human subjects has been validated by the ability of exendin 9-39, a GLP-1R antagonist, to block GLP-1RA-i nduced glucose-stimulated insulin secretion from human islets, and after a 150 g glucose load in healthy human volunteers.^27^ In adults with T2D, liraglutide improves beta cell function, as evident by the increase in first-and second-phase insulin secretion.^28^

In addition to its incretin effect, GLP-1 also suppresses glucagon secretion from alpha cells. GLP-1-mediated inhibition of glucagon secretion occurs through three pathways: (1) GLP-1 stimulates the secretion of somatostatin, a potent suppressor of glucagon;^29^ (2) inhibition of glucagon secretion can also occur through a PKA-d ependent pathway;^30^ and (3) GLP-1-stimulated insulin secretion from beta cells indirectly suppresses glucagon secretion from alpha cells. In response to glucose loading, individuals with T2D display delayed insulin secretion as well as failure of glucagon suppression, contributing to postprandial hyperglycaemia.^31^ GLP-1-mediated suppression of glucagon secretion is thus expected to augment its insulinotropic effect in normalizing postprandial hyperglycaemia.^32^

Early rodent studies demonstrating GLP-1RA treatment expansion of the beta cell mass generated excitement about its potential to reverse T2D in humans.^22^ Subsequent work showed that GLP-1R-mediated stimulation of beta cell proliferation only occurred in young but not older rodents.^33^ In human subjects, while GLP-1RAs may improve beta cell function during treatment, there is no clinical evidence of sustained improvement after treatment discontinuation in either adults or adolescents.^34–37^

As GLP-1RAs stimulate insulin secretion, the question of its potential to promote beta cell exhaustion has also been raised. Prior studies have shown that patients treated with sulfonylureas are at increased risk for beta cell functional decline.^38^ In a humanized mouse model using transplanted islets, daily injection of high-dose liraglutide for more than 200 days resulted in beta cell dysfunction.^39^ To date, there have been no clinical studies demonstrating adverse impact of GLP-1RAs on beta cell function.

**Table 1:** Summary of paediatric clinical trial results of glucagon-like peptide-1 receptor agonist use in paediatric type 2 diabetes

**Study**	**Design**	**Sample size**	**Age in years**	**Main inclusion criteria**	**Dose**	**Duration**	**Efficacy**	**Safety**
**Liraglutide**							
Klein et al. (2014)^65^	RCT	21	10-17	HbA1C 6.5-11.0% With lifestyle modifications alone or with metformin, and BMI >85^th^ percentile	1.8 mg DE: 0.3 mg, 0.6 mg, 1.2 mg, 1.8 mg	5 weeks	HbA1c change: ETD -0.9%, favouring intervention (p=0.0007)	Mild AEs in 71.4% of intervention (57.1% with Gl AEs) versus mild AEs in 42.9% of placebo (28.6% with Gl AEs) Transient Gl symptoms = most common AE, during titration No SAEs
Tamborlane et al. (2019)^6^	RCT	135	10-16	HbA1c 7.0-11.0% if lifestyle modifications alone or 6.5-11.0% if metformin ± insulin, and BMI >85^th^ percentile	1.8 mg DE: 0.6 mg, 1.8 mg	26 weeks + 26 weeks open label	HbA1c change: ETD of -1.06% favouring intervention (p<0.001) Difference increased to -1.30 after 52 weeks Secondary endpoints: HbA1c <7.0%: 63.7% in intervention versus 36.5% in placebo (p<0.001) Intervention group had decrease in fasting plasma glucose level at both time points, compared with increase in placebo	AEs in 84.8% of intervention versus 80.9% of placebo Rate of AE per 1 patient-year of exposure was higher with intervention than with placebo Gl AEs = most common in intervention, mostly during initial 8 weeks, mild, resolved Mild hypoglycaemia more frequent in intervention (24.2%) versus placebo (10.3%), no severe hypoglycaemia in intervention Lipase levels higher in intervention (treatment ratio 1.20), amylase levels similar SAEs more frequent in intervention (13.6%) versus placebo (5.9%)
**Exenatide**								
Malloy et al. (2009)^66^	RCT, crossover	13	10-16	HbA1c 6.0-11.0%, lifestyle modifications, metformin, or sulphonylurea therapy, and body weight of >50 kg	Three single doses of 2.5 pg or 5 pg, preprandial	Up to 5 weeks	In both dose groups: Reduction in postprandial blood glucose level compared with placebo (p<0.01) Reduction in postprandial glucagon levels compared with placebo (p<0.01)	AEs in 15% of intervention (2 subjects), AE in one subject was unrelated to study drug No severe or symptomatic hypoglycaemia
Tamborlane et al. (2022)^5^	RCT	83	10-17	HbA1c 6.5-11.0% if not taking insulin or sulphonylurea, HbA1c 6.5-12.0% if taking insulin or sulphonylurea	2 mg extended-release, weekly	24 weeks + 28 weeks open label	HbA1c change: ETD of -0.85%, favouring intervention group (p=0.012)	AEs and SAEs less in intervention group: 61.0% of intervention versus 73.9% of placebo; 3.4% of intervention versus 4.3% of placebo, respectively Most Gl AEs were mild or moderate and resolved No major hypoglycaemic events in either group
**Dulaglutide**								
Arslanian et al. (2022)^7^	RCT	154	10-17	HbA1c 6.5-11.0% if taking metformin ± insulin or HbA1c6.5-9.0 if lifestyle modifications alone, and BMI >85^th^ percentile, and body weight >50 kg	0.75 mg or 1.5 mg	26 weeks + 26 weeks open label	HbA1c change: -0.6% in the 0.75 mg group and -0.9% in the 1.5 mg group (p<0.001 for both groups versus placebo) ETD of -1.4 for pooled intervention groups versus placebo Secondary endpoints: 51% in intervention with HbA1c of <7.0% versus 14% in placebo (p<0.001) Fasting glucose decreased in the pooled dulaglutide groups by -18.9 mg/dL (p<0.001) Cardiometabolic findings: LDL,TG decreased in intervention	AEs in 73-75% intervention versus 69% in placebo; SAEs in 2% intervention versus 6% placebo. Gl AEs = most frequent, more in intervention group, mostly mild, mostly transient within 2 weeks of initiation Hypoglycaemia, injection site reaction, hypersensitivity, pancreatic enzyme level, calcitonin level did not differ between groups

*All of the above medications have received United States Food and Drug Administration approval for type 2 diaPetes in age 10 years and above.*

*AE = adverse event; BMI = body mass index; DE = dose escalation; ETD = estimated treatment difference; Gl = gastrointestinal; HbA 1 c = haemoglobin A1 c; LDL = low-density lipoprotein; RCT= randomized controlled trial; SAE = serious adverse event; T2D = type 2 diabetes; TG = triglycerides.*

### Management of obesity

In addition to its insulinotropic actions, GLP-1RAs are also effective agents in promoting weight loss. Although distension of the proximal gut activates GLP-1R-expressing afferent sensory vagal neurones to inhibit gastric emptying and small intestinal motility, this pathway does not mediate GLP-1RA-driven weight loss. Downregulation of GLP-1R in the nodose ganglion in rodents prevented GLP-1RA-i nduced inhibition of gastric emptying but had no effect on food intake or body weight.^21^

Several lines of evidence support the brain as the central target of GLP-1RA-i nduced weight loss. Injection of liraglutide or exendin-4 peripherally, activated multiple nuclei in the hindbrain and hypothalamus (periventricular nucleus, area postrema and NTS).^40,41^ In rats, intracerebroventricular injection of the GLP-1R inhibitor, exendin 9-39, increased food intake.^42–44^ Postnatal knockdown of hypothalamic *Glp1r* (the gene encoding GLP-1R) expression increased food intake and body weight.^22^ In addition to its effect on homeostatic feeding, GLP-1RAs also activate brain areas in the mesolimbic system to suppress reward behaviour and palatability.^22^ The combination of GLP-1 action on homeostatic and hedonic feeding likely contributes to its appetite suppressant and weight loss effect.

Despite the unequivocal pharmacological effect of GLP-1 RAs, the mechanism of physiological central nervous system (CNS) GLP-1R signalling remains incompletely understood. Although peripherally injected GLP-1RAs can cross the blood–brain barrier,^22,45,46^ it is unclear if endogenous GLP-1 activates central GLP-1R signalling. The pattern of neuronal activation differed between mice (or rats) that received injection of long-acting GLP-1RAs peripherally and intra-portal vein injection of native GLP-1.^22,47^ Given the short half-l ife of native GLP-1 (less than 2 minutes), it is questionable whether peripherally or centrally derived GLP-1 activates CNS GLP-1R signalling. Finally, as mechanistic studies of CNS GLP-1R signalling largely comes from rodent models, the physiological importance of CNS GLP-1R signalling in weight control in humans remains unknown.

### Management of diabetic nephropathy

The TODAY study demonstrated that diabetic nephropathy (DN) occurs early in youth-onset T2D, with a baseline prevalence of 8% at study entry (mean diabetes duration of 7.8 months) and a cumulative incidence of 54.8% after a mean diabetes duration of 13.3 years.^48^ Treatment for DN in children consists of glycaemic management, lifestyle modification and the use of angiotensin-c onverting enzyme (ACE) inhibitors.^8,49^ Studies of adults with T2D have demonstrated that GLP-1RAs delay the progression of diabetic kidney disease.^50–52^ *In vitro* and *in vivo* animal models point to two GLP-1RA-mediated pathways. GLP-1RAs may promote natriuresis by inhibiting the Na^+^/H^+^ exchanger isoform 3 on the apical membrane of the proximal tubule.^53^ GLP-1RAs also inhibit angiotensin II (ANG II) action at different levels of the renin–angiotensin pathway. GLP-1RAs have been shown to increase ACE2 expression and activity, and downregulate the expression of ANG II production.^54^ It may also protect the kidneys by limiting the ANG II-i nduced tissue fibrosis by altering the expression of its receptor subtypes, ANG II receptor type 1 and ANG II receptor type 2.^55^ Finally, GLP-1 may lower ANG II-i nduced oxidative stress in a PKA-dependent pathway.^54^ Future trials testing the efficacy of GLP-1RAs on the prevention of DN in youth-onset T2D is needed to expand management strategies for this vulnerable population.

### Other metabolic effects

Elevated alanine aminotransferase – a marker recommended for screening of nonalcoholic fatty liver disease – has been found in 48% of patients with paediatric T2D.^56,57^ GLP-1RAs have been shown to improve hepatic steatosis in adults who are overweight or have T2D or polycystic ovary syndrome.^58–62^ GLP-1RA-mediated weight loss may decrease lipotoxicity, and enhanced hepatic insulin sensitivity may improve hepatic mitochondrial function.^62^ Additionally, cardiovascular risk factors such as hypertension, dyslipidaemia and chronic inflammation are common in youth-onset T2D.^48^ Sixteen per cent of the TODAY study participants had abnormal left ventricular structure.^63^ GLP-1RAs appear to exert cardioprotective effects by stabilizing atheroma via improvement of endothelial function and reduction of vascular wall inflammation.^64^ Although serious cardiovascular events remain rare in young adults with youth-onset T2D, the case rates will likely increase as the incidence of T2D continues to rise. Future studies are needed to determine if GLP-1RA use will similarly reduce major atherosclerotic cardiac events.

## Safety and efficacy of glucagon-like peptide-1 receptor agonists in paediatric type 2 diabetes

Currently, there are three GLP-1RAs approved for the treatment of T2D in youth aged 10 years and older, including liraglutide, extended-release exenatide and dulaglutide (*Table 1*). Since the approval of metformin in 2000, liraglutide (1.8 mg daily) was the first non-i nsulin medication to receive FDA approval in 2019 for the treatment of T2D in youth. A randomized, placebo-controlled trial was conducted in 2014 to assess the pharmacokinetics, pharmacodynamics and safety of liraglutide in 21 youth aged 10–17 years with T2D.^65^ A dose-dependent improvement in HbA1c was observed in this small cohort. Pharmacokinetics and side effects were comparable to that observed in adults. Given these findings of efficacy, tolerability and safety, a subsequent randomized, placebo-controlled study of 135 youth aged 10–16 years with T2D was conducted by the Ellipse Trial Investigators in 2019.^6^ Liraglutide was started at 0.6 mg daily and titrated up to 1.8 mg daily for 26 weeks, followed by a 26-week open-l abel extension period. At 26 weeks, there was an estimated treatment difference in HbA1c change from baseline of -1.06% (p<0.001) favouring the liraglutide group, which increased to -1.30% after 52 weeks. HbA1c levels of <7.0% were achieved in 63.7% of the liraglutide group versus 36.5% of the placebo group (p<0.001), and the liraglutide group had a decrease in fasting plasma glucose level at both time points. In contrast, there was an increase in the placebo group.

Exenatide extended-release was then approved in 2021 for youth with T2D aged 10 years and older, which is the first long-acting GLP-1RA approved for adolescents, given at a dose of 2.0 mg weekly.^5^ An initial randomized, placebo-controlled, crossover study on the pharmacology and tolerability of 2.5 μg and 5 μg exenatide pre-prandial injections in 13 adolescents with T2D demonstrated a significant reduction in postprandial blood glucose level and glucagon secretion.^66^ Subsequently, 83 adolescents aged 10–17 years were randomized to receive exenatide 2 mg weekly for 24 weeks, followed by a 28-week open-l abel period.^5^ At 24 weeks there was a significant between-group difference in HbA1c change of -0.85%, favouring the treatment group. Mean difference in fasting glucose, systolic blood pressure and triglyceride levels also favoured the treatment group, although it did not reach significance. Adverse events occurred in 61.0% of the treatment group compared with 73.9% of the placebo group, and no major hypoglycaemic events occurred in either group.

**Table 2:** Summary of paediatric clinical trial results of glucagon-like peptide-1 receptor agonist use in paediatric obesity

**Study**	**Design**	**Sample size**	**Age in years**	**Main inclusion criteria**	**Dose**	**Duration**	**Efficacy**	**Safety**
**Liraglutide**								
Kelly etal. (2020)^63^	RCT	251	12-17	BMI >95^th^ percentile, did not exclude if T2D	3.0 mg daily	56 weeks + 26 weeks follow up	zBMI change (SD): ETD of -0.22, favouring intervention (p=0.002) Reduction in BMI of >5%: 43.3% of intervention versus 18.7% of placebo Reduction in BMI of >10%: 26.1% of intervention versus 8.1% of placebo	Gl AEs more frequent in intervention (64.8% versus 36.5%) AEs leading to discontinuation more frequent in intervention (10.4% versus 0.0%) Few SAEs (2.4% versus 4.0%)
Bensignor et al. (202 D^69^	RCT	134	10-16	BMI >85^th^ percentile andT2D	0.6 mg daily 1.2 mg daily 1.8 mg daily	52 weeks	BMI change: ETD of -0.89 (kg/m^2^), favouring intervention (p=0.036) % change in BMI: ETD of -2.73%, favouring intervention (p=0.028) %BMIp95 change: ETD of -4.42%, favouring intervention (p=0.038) Findings are significant at 52 weeks, not at 26 weeks	Not evaluated
**Exenatide**								
Kelly etal. (2012)^70^	Randomized, open-label, crossover	12	9-16	BMI >1.2 times the 95^th^%, or BMI >35 kg/m^2^	10 pg twice daily DE: 5 pg twice daily, 10 pg twice daily	3 months	BMI change: ETD of -1.71 (kg/m^2^), favouring intervention (p=0.01) % change in BMI: ETD of -4.92%, favouring intervention (p=0.009) Total body weight change: ETD of -3.9 kg, favouring intervention (p=0.02) Insulin-related findings: Fasting insulin change: ETD of -7.5 mU/L, favouring intervention (p=0.02) Insulin sensitivity: ETD of +6.1, favouring intervention (p=0.02) p-cell function: ETD of +17.97, favouring intervention (p=0.03)	Mild nausea in 36%, vomiting in 27%, headache in 27%, abdominal pain in 27%, injection site bruising in one participant No hypoglycaemia or pancreatitis
Fox etal. (2022)^71^	RCT	100	12-18	BMI >1.2 times the 95^th^ percentile	2.0 mg extended release, weekly	52 weeks	% change in BMI: ETD of -4.1%, favouring intervention, did not reach significance (p=0.078) Cardiometabolic findings: TG/HDL ratio: ETD of -0.61, favouring intervention (p=0.05)	AE frequency similar between groups (96.9% of intervention versus 90.9% of placebo) Gl AEs more common in intervention No serious adverse event directly related to the study drug
**Semaglutide**								
Weghuber et al. (2022)^72^	RCT	201	12-17	BMI >95^th^ percentile or BMI >85^th^ percentile + weight-related coexisting condition	2.4 mg weekly	68 weeks	% change in BMI: ETD of -16.7%, favouring intervention group (p=<0.001) Weight loss of >5%: 73% of intervention versus 18% of placebo Cardiometabolic findings: Improved waist circumference, HbA1c, lipids, AST were greater in intervention	Gl AEs greater (62% of intervention versus 42% of placebo) 4% with cholestasis in intervention SAEs in 11% of intervention versus 9% of placebo

*Liraglutide and semaglutide have received United States Food and Drug Administration approval for oPesity in age 12 years and above.*

*AE = adverse event; AST = aspartate transaminase; BMI = body mass index; %BMip95 = body mass index per cent over the 95th percentile; DE = dose escalation; ETD = estimated treatment difference; Gl = gastrointestinal; HbA 1 c = hemoglobin A1 c; HDL = high-density lipoprotein; p85 = 85th percentile; RCT = randomized controlled trial; SAE = serious adverse event; SD = standard deviation; T2D = type 2 diabetes; TG = triglyceride; zBMI = body mass index Z-score.*

Dulaglutide is the newest GLP-1RA approved by the FDA for adolescents 10 years and older with T2D. A randomized, placebo-controlled trial was completed by the Assessment of Weekly Administration of LY2189265 in Diabetes-Pediatric Study (AWARD-PEDS) investigators with 154 youth aged 10–17 years of age who received weekly injections of dulaglutide at a dose of either 0.75 mg, 1.5 mg or placebo for 26 weeks, followed by a 26-week open-l abel phase.^7^ At 26 weeks, there was a significant decrease in mean HbA1c of -0.6% in the 0.75 mg group and -0.9% in the 1.5 mg group (p<0.001 for both groups versus placebo). HbA1c levels of <7.0% were achieved in 51% of the pooled dulaglutide groups compared with 14% of the placebo group (p<0.001), and fasting glucose concentration also significantly decreased in the pooled dulaglutide groups (-18.9 mg/dL, p<0.001). Adverse events were similar between placebo and treatment arms (69%, 75% and 73% in the placebo, 0.75 mg and 1.5 mg arms, respectively). Consistent with adult data, gastrointestinal adverse events (nausea, vomiting, diarrhoea) were more frequent in the dulaglutide group compared with the placebo group; the symptoms were mostly mild and were transient within 2 weeks of initiation. The study concluded that in youth with T2D, dulaglutide taken once weekly (0.75 mg or 1.5 mg) improved glycaemic control compared with placebo.

Contrary to findings from adult studies, the pivotal GLP-1RA paediatric T2D trials do not demonstrate significant improvement in weight loss. Adolescents with T2D display more insulin resistance and glycaemic failure compared with adults.^1,19^ GLP-1RA treatment of adults with T2D resulted in less weight loss compared with participants without T2D.^67^ The AWARD-PEDS investigators thus speculated that the catabolic effect of hyperglycaemia in the placebo group may obscure detection of weight loss in the dulaglutide-treated groups. Although the mechanism is unknown, it is possible that the insulin resistance observed during adolescence may also contribute to the relative resistance to GLP-1RA-mediated weight loss.

## Safety and efficacy of glucagon-like peptide-1 receptor agonists in paediatric obesity

Following the observed benefit of higher GLP-1RA doses on weight loss in adults, multiple studies have been conducted evaluating GLP-1RAs specifically for obesity in the adolescent population (*Table 2*).^68–72^ A study was completed in 2012 evaluating daily exenatide for weight loss in youth 9–16 years of age, with dose increased from 5 μg twice daily to 10 μg twice daily over a 3-month period.^70^ Body mass index (BMI) (-1.7 kg/m^2^, p=0.01) and total body weight (-3.9 kg, p=0.02) both decreased significantly. However, immediate-release exenatide has not received FDA approval for the indication of paediatric obesity. A subsequent study showed that extended-release exenatide may partially reduce the risk of BMI rebound in adolescents with severe obesity who achieved weight loss with dietary interventions.^71^ Currently, liraglutide (3 mg daily) is approved for youth aged 12 years and older with obesity, as a supplemental indication for chronic weight management. In a study of 251 adolescents aged 12–17 years, liraglutide 3.0 mg daily was superior to placebo in terms of BMI Z-score change from baseline at 56 weeks, with an estimated treatment difference of -0.22 (p=0.002).^68^ A reduction in BMI of at least 5% was observed in 43.3% of the liraglutide group versus 18.7% in the placebo group, and a reduction in BMI of at least 10% was observed in 26.1% versus 8.1% in these groups, respectively. There was an estimated difference of -4.6% in absolute BMI and of -4.50 kg (-5.0%) in body weight, favouring liraglutide. Reductions in absolute BMI and per cent change in BMI were also seen in youth with obesity and T2D, when taking liraglutide for 52 weeks.^69^

More recently, the efficacy of an additional extended-release GLP-1RA, semaglutide, was studied in adolescents with obesity.^72^ Semaglutide at a dose of 2.4 mg weekly is approved for adults with obesity, or who are overweight and have weight-related coexisting conditions, for longterm weight management as an adjunct to lifestyle modifications. In the recent randomized, placebo-controlled study of 201 youth aged 12–17 years taking semaglutide at a dose of 2.4 mg weekly for 68 weeks, there was an estimated difference of -16.7% in mean change in BMI from baseline, favouring the semaglutide group (p<0.001). Weight loss of 5% or more was seen in 73% of the treatment group versus 18% in the placebo group. Improvement in cardiometabolic risk factors (waist circumference, lipids [except high-density lipoprotein cholesterol] and alanine aminotransferase) were also greater with semaglutide compared with placebo. Semaglutide 2.4 mg recently received FDA approval for the treatment of obesity in children 12 years and older.

## Precautions with glucagon-like peptide-1 receptor agonists

Similar to adults, the most common side effects of GLP-1RAs in adolescents are gastrointestinal symptoms. GLP-1RAs should be initiated at a low dose, with a gradual titration to limit side effects and increase tolerability. The length of titration depends on the formulation. The use of GLP-1RAs is contraindicated in patients with a personal or family history of medullary thyroid carcinoma or in patients with multiple endocrine neoplasia type 2. Early concerns of possible pancreatitis risk have not been corroborated by more recent reports, although pancreatitis remains a contraindication to GLP-1RA use.^73^ In patients with suspected pancreatitis, GLP-1RAs should be discontinued and should not be resumed if the diagnosis is confirmed. Due to the risk for dehydration and acute kidney injury, patients who report gastrointestinal symptoms and have renal impairment should have monitoring of their renal function.

## Barriers to glucagon-like peptide-1 receptor agonist use

Despite the FDA approval of liraglutide in 2019 for paediatric T2D, the uptake of GLP-1RAs in this population has been slow. Gourgari et al. assessed clinicians’ experience with prescribing GLP-1RA medications.^74^ Members of the Pediatric Endocrine Society (n=102) completed a survey evaluating barriers and perceived outcomes. The primary intentions of GLP-1RA use were to lower HbA1c levels (88%) and for weight management (82%). Alternative indications included reduction of total daily insulin dose and the treatment of comorbidities such as nonalcoholic fatty liver disease, hypertension and irregular menses. In those who did not prescribe GLP-1RAs, lack of clinical experience was a primary barrier (42%, p=0.0024), especially in those with more than 5 years of clinical experience. The majority initiated a GLP-1RA after unsuccessful treatment with metformin. About two-thirds of clinicians prescribed these medications in youth 12 years and older, while others (19%) initiated in those below 12 years of age. Liraglutide was the GLP-1RA of choice in the majority of cases (93%, p<0.0001).

Beyond prescription pattern, GLP-1RA access may also be limited due to cost. As a class, the high cost of these medications contributes to limited insurance coverage of these medications, especially for the indication of obesity.^75^ The above clinician experience assessment by Gourgari et al. was conducted in the United States, where at least some proportions of patients were able to obtain insurance authorization. However, it is important to note that GLP-1RAs are typically not an insurance-c overed benefit in developing countries. The average monthly cost of these medications in the United States ranges from US$930 for dulaglutide to US$1,349 for semaglutide and liraglutide, with annual costs ranging from US$11,000 to US$16,000.^76,77^ Even when covered by insurance, the out-of-pocket expense may be cost prohibitive.

## Glucagon-like peptide/glucose-dependent insulinotropic polypeptide dual agonist therapy

Glucose-dependent insulinotropic polypeptide (GIP) is another gut-secreted hormone with a dominant insulinotropic effect. Physiologically, it is secreted from the K cells in the duodenum and jejunum.^78^ In the adult population, tirzepatide, which is a GLP-1/GIP dual agonist, was demonstrated to be even more effective than GLP-1RA monotherapy. Studies in adults have shown that tirzepatide is superior to placebo, GLP-1RAs and basal insulin in terms of glycaemic control and body weight reduction. Data are currently lacking in the paediatric population, and further studies are needed to support its potential use.

## Future directions

Despite clinical guidelines recommending a 5–10% weight reduction for youth-onset T2D, there remains a gap regarding recommendations for utilization of novel agents that target the multifaceted pathophysiology of T2D in this age group. The recent FDA approval of several GLP-1RAs for paediatric T2D and obesity heralds a much-welcomed increase in the armamentarium for the management of these conditions. Much work is needed, however, to expand the use of GLP-1RAs in children.

Many barriers exist in gaining paediatric approval for medications already approved for adult use. The FDA mandates phase III clinical trials for all new medications for each clinical indication to protect patient safety. Completion of such trials in adolescents with T2D is difficult due to a relatively small patient population (compared with adults), stringent exclusion criteria, and the difficulty of participants/caregivers repeatedly taking time off from school and work.^79^ Families of historically marginalized races and ethnicities may also be reluctant to participate in clinical trials. Pragmatic designs are needed to allow trials to augment enrollment and overcome historical barriers. These strategies may include involvement of pertinent stakeholders in trial design, recruitment and execution.^80^ In addition, long-term follow-up studies will be needed to determine if GLP-1RAs render the same cardio-and renal-p rotective benefits in youth-onset T2D.

There may also need to be a shift in the paradigm in balancing conducting rigorous safety and efficacy trials of novel agents in paediatric cohorts, against prescribing these agents off-l abel when available evidence supports a substantial potential health benefit. For many clinicians, the lack of safety and efficacy data impacts prescribing practices and agent selection. Although sparse, the randomized controlled trials that do exist in youth support the efficacy and safety of GLP-RAs in this population. One might argue that once there is FDA approval of one agent in a class of medications, in the context of safety and efficacy data of other agents in the class among adults, the initial data are sufficient to begin the utilization of that agent if the benefit outweighs the risk. Consideration needs to be given to the health outcome of adolescents who *cannot* benefit from novel medications, due to the length of time required to conduct clinical trials in a relatively small patient population. Specifically, for GLP-1RAs, their mechanism of action may provide an opportunity to not only target weight reduction and glycaemic control, but also reduce cardiovascular, renal and hepatic risk factors, all of which could significantly improve the health trajectory of youth with T2D and associated microvascular and macrovascular complications. Taken together, the pharmacotherapy profile for GLP-1RAs is very favourable for youth-onset T2D. However, high cost and inadequate insurance coverage may continue to limit access to these agents in paediatric cohorts. Further investigation both at the policy and clinical guideline levels must be conducted to support an equitable and accessible roll-out of these agents into paediatric clinical care.
